# Perceptions of health care professionals on the safety and security at Odi District Hospital, Gauteng, South Africa

**DOI:** 10.4102/phcfm.v9i1.1441

**Published:** 2017-10-27

**Authors:** Sunday O. Okeke, Langalibalele H. Mabuza

**Affiliations:** 1 Department of Family Medicine & Primary Health Care, Sefako Makgatho Health Sciences University, South Africa

## Abstract

**Background:**

For optimum delivery of service, an establishment needs to ensure a safe and secure environment. In 2011, the South African government promulgated the National Core Standards for Health Establishments for safety and security for all employees in all establishments. Little is known about whether these standards are being complied to.

**Aim and setting:** To assess the perceptions of health care professionals (HCPs) on safety and security at Odi District Hospital.

**Methodology:**

A sample of 181 out of a total of 341 HCPs was drawn through a systematic sampling method from each HCP category. Data were collected through a self-administered questionnaire. The SPSS® statistical software version 22 was used for data analysis. The level of statistical significance was set at < 0.05.

**Results:**

There were more female respondents than male respondents (136; 75.10%). The dominant age group was 28–47 years (114; 57.46%). Perceptions on security personnel, their efficiency and the security system were significantly affirmed (*p* = 0.0001). The hospital infrastructure, surroundings and plan in emergencies were perceived to be safe (*p* < 0.0001). The hospital lighting system was perceived as inadequate (*p* = 0.0041). Only 36 (20.2%) HCPs perceived that hospital authorities were concerned about employees’ safety (*p* < 0.0001).

**Conclusion:**

HCPs had positive perceptions regarding the hospital’s security system. Except for the negative perceptions of the lighting system and the perceived lack of hospital authorities’ concern for staff safety, perceptions of the HCPs on the hospital working environment were positive. The hospital authorities need to establish the basis of negative perceptions and enforce remedial measures to redress them.

## Background

Health care institutions are meant to provide a safe and secure environment for all users of the facilities.^1^ Yet threats to the safety and security of patients and hospital staff continue to affect the physician’s oath to ‘do no harm’,^2^ in the sense that patient care may be compromised where health care professionals (HCPs) become hesitant to offer help for fear of endangering their own lives.

In British Columbia (BC), the health care sector accounted for more injuries and time loss than any other sector until 2003. However, according to the Workers Compensation Board (WCB) of BC, the injury rate in the BC health care sector has declined dramatically since 1998.^3^ The WCB examined how this was accomplished and linked it to the manner in which occupational health and safety measures were introduced through implementation of safety protocols in the interests of patient safety^4^; Kjellén defined ‘safety’ as protection against hazards, whereas security is protection against threats.^5^ The New Oxford Dictionary of English^6^ describes safety and security in the following ways: ‘safety’ is a condition of being protected from risk or injury, denoting something designed to prevent injury or damage, for example, safety barriers. ‘Security’ is a state of being free from danger or threat, for example, procedures followed or measures taken to ensure that state of feeling stable and free from fear or anxiety.^7^

From the above narrative, it becomes clear that the difference between security and safety is not remarkably sharp. Both are conditions where one is well protected and without risks. However, the condition of safety is about being protected, while the condition of security is about being free from danger.^7^

Therefore, according to the aforesaid explanations, it can be said that ‘security’ entails the systems, personnel and processes employed to impart the feeling of ‘being secure’ in an establishment, whereas ‘safety’ refers mainly to the physical infrastructure, surroundings, installations, plans and protocols put in place to eliminate threats or dangers, for example, ‘being put in a place of safety’. Nevertheless, it must also be noted that safety cannot exist without security, and vice versa.

The South African Medical Research Council (MRC) reported that crime and violence impacted on health care service delivery in the Western Cape where it was found that 60% of the HCPs had to deal frequently with workplace crime and violence, with 92.3% reporting verbal abuse and 36.4% threats of assault.^8^

In 2001, a hospital ethics audit was conducted at Chris Hani Baragwanath Hospital (CHBH) one of the objectives of which was to assess its general working environment and the possible effects that the environment might have on personal and interpersonal behaviour of the employees. It found that workplace violence was rife; two-thirds of the staff affirmed that the number of security staff was inadequate and 76% felt that the security personnel were poorly equipped to do their job. A total of 57% believed that the screening of visitors was deficient and that, in general, there was a huge lack of confidence in the capacity and ability of security staff to ensure a safe environment.^9^

The South African situation discussed above has indicated that the country is faced with a high violence rate from which health care institutions are not exempt. However, the perceptions of the HCPs who are frequently at the receiving end of violence threatening their security and safety is unknown in their places of work. There is a scarcity of literature information relating to perceptions of HCPs about safety and security in health care institutions within South Africa and beyond. Studies on some aspects of safety and security have been conducted abroad.^3,10,11,12,13^ Notably, most of these studies assessed the safety and security in health care institutions indirectly, for example, assessing workplace violence against health workers.^14,15,16,17^ This study is conducted with the hope of gaining deep insight into the perception of HCPs regarding their security and safety in a typical district hospital in South Africa.

## Research methods and design

### Study setting

The study was conducted among HCPs at Odi District Hospital, Gauteng Province.

### Study design

This was a descriptive cross-sectional study

### Study population

All the 341 HCPs working at Odi District Hospital at the time of the study constituted the study population. They included 23 full-time and 9 part-time doctors, 7 family medicine registrars, 266 nurses, 12 pharmacists, 12 radiographers, 2 physiotherapists, 2 social workers, 2 clinical associates, 2 dieticians, 1 speech therapist, 1 occupational therapist and 2 clinical psychologists.

### Sampling procedure

At the confidence level of 95%, confidence interval of 5% and a target population of 341, the sample size worked out to be 181. To obtain a representative sample from each category of HCPs, a *pro rata* number for the particular category was used. Therefore, each category group was represented by 181/341 (0.53) used to systematically select the individual respondents. However, where a category was represented by only one individual that individual was included in the sample. In summary, the sample included 12 full-time doctors, 5 part-time doctors, 4 family medicine registrars, 141 nurses, 6 pharmacists, 6 radiographers, 1 physiotherapist, 1 social worker, 1 clinical associate, 1 dietician, 1 speech therapist, 1 occupational therapist and 1 clinical psychologist – a total of 181 health care professionals (Table 3).

### Data collection

Section 7.3 of the National Core Standards for Health Establishments in South Africa deals with the safety and security of establishments. Subsection 7.3.1 states that ‘People and property should be actively protected to minimise safety and security risks’.^18^ Seven criteria are stipulated for the achievement of this ideal, namely:

- Security systems to safeguard the building, patients, visitors and staff.
- The layout and security systems for the protection of vulnerable patients.
- Internal and external lighting to be adequate to protect patients, visitors and staff.
- All security incidents to be reported and addressed accordingly.
- Awareness of safety and security issues to be promoted to the staff.
- There should be an up-to-date documented certification from the local fire authority which verifies that the health establishment complies with relevant fire safety regulations.
- An emergency plan is to be made available indicating that patient well-being is at all times protected.

In keeping with Section 7.3 of the National Core Standards for Health Establishments in South Africa, the above-mentioned definitions and explanations, assessment of HCPs’ perceptions on security and safety can be categorised according to the following parameters:^6,7^

- Parameters on security:
  ■ presence of security personnel
  ■ security personnel efficiency
  ■ the security system (including incident reporting and processing of reported incidents).
- Parameters on safety:
  ■ hospital infrastructure and surroundings
  ■ safety from fire
  ■ the hospital lighting system
  ■ the emergency evacuation plan (and staff confidence to follow it)
  ■ safety from possible harm from patients and their visitors
  ■ the protocol on violence prevention in the hospital
  ■ the hospital authorities’ concern for employees’ safety.

A pilot study was conducted at another district hospital (Jubilee Hospital in Hammanskraal) where 15 HCPs completed the questionnaire. This was done to refine the questionnaire and eliminate possible ambiguities in the questions. The self-administered questionnaire was created de novo by the research team, with the assistance of a statistician, and was distributed by the researcher and two trained research assistants to a consenting respondent. All completed questionnaires were collected for analysis. As respondents of each category were determined by the pro rata percentage, randomisation in each category was achieved by systematic sampling whereby respondents were selected from a sample of numbers allocated to each HCP in that category – as described in the sampling procedure above.

### Data analysis

Descriptive data were presented as frequencies, tables and bar diagrams where applicable. Analysis was done using SPSS^®^ statistical software version 22. Univariate analyses of the baseline characteristics and bivariate statistical analyses of dependent and independent variables for associations using the chi-square test, where applicable, were done. The statistical level of significance was set at < 0.05.

### Ethical considerations

Ethical clearance for the study was obtained from Medunsa Research Ethics Committee (MREC) of the University of Limpopo, now Sefako Makgatho Health Sciences University (SMU). The clearance certificate number is MREC/M/19/2014:PG. The senior clinical manager of Odi District Hospital, Gauteng Province, gave permission for the study to be conducted at that hospital. Written informed consent was obtained from all the respondents using the University of Limpopo (Medunsa campus) consent forms before participation. Respondents were informed of their right to withdraw from the study at any stage. Confidentiality and anonymity of the respondents were maintained during and after the study. Data were analysed as group data and no personal identifiers were reflected in the data collection forms.

## Results

### Baseline characteristics

Table 1 shows the participants’ baseline characteristics. Of the 181 participants, there were more females (136; 75.1%) than males (45; 24.9%). Most of the participants (63%) were aged between 28 and 47 years. Approximately 3% were above 58 years. The majority were single (94; 51.90%). Table 2 outlines the HCPs’ professional categories. The majority were nurses (141; 77.9%) followed by doctors (21; 11.6%).

**TABLE 1:** Baseline characteristics (*n* = 181).

Characteristics	Frequency	Percentage
**Gender**		
Female	136	75.1
Male	45	24.9
**Total**	**181**	**100**
**Ages**		
18–27	32	17.68
28–37	72	39.78
38–47	42	23.20
48–57	30	16.57
58–67	5	2.76
**Total**	**181**	**100**
**Marital status**		
Single	94	51.93
Married	76	41.99
Widow	4	2.21
Widower	3	1.66
Divorcee	4	2.21
**Total**	**181**	**100**

**TABLE 2:** Healthcare professional categories (*n* = 181)

Health care professional	Sample frequency	Percentage
Doctors:	10	5.50
Full-time medical officers	5	2.80
Sessions doctors	4	2.20
Family medicine registrars Community service doctors	2	1.10
**Total**	**21**	**11.60**
Nurses:		
Professional nurses	75	41.40
Enrolled nurses	32	17.70
Nursing assistants	34	18.90
**Total**	**141**	**77.90**
Radiography	6	3.31
Pharmacist	6	3.31
Dietician	1	0.55
Social worker	1	0.55
Physiotherapist	1	0.55
Clinical associate	1	0.55
Psychologist	1	0.55
Speech therapist	1	0.55
Occupational therapist	1	0.55
**Total**	**181**	**100.00**

### Perceptions on security

Table 3 shows that perceptions on security conferred by security personnel presence, efficiency of security personnel and efficiency of the security system were significantly affirmed (*p* = 0.0001). However, there was no difference between those affirming versus those dissenting regarding the incident reporting system to the relevant hospital authorities and the efficiency of the authorities (in dealing with reported security incident): *p* = 0.2086 and *p* = 0.2740 respectively.

**TABLE 3:** Perceptions of all health care professionals on security.

Perception	Agree *n* (%)	Disagree *n* (%)	Do not know *n* (%)	Agree versus disagree *P*-value
Security conferred by the presence of security personnel (*n* = 181)	101 (55.8)	59 (32.6)	21 (11.6)	*p* < 0.0001
Security personnel efficiency (*n* = 179)	97 (54.2)	58 (32.4)	24 (13.4)	*p* < 0.0001
Efficiency of the security system in protecting patients and staff (*n* = 181)	95 (52.5)	66 (36.5)	20 (11.0)	*p* = 0.0013
Incident reporting system to the relevant authority (*n* = 181)	67 (37.0)	57 (31.5)	57 (31.5)	*p* = 0.2086
Efficiency of hospital authorities in dealing with reported security incidents (*n* = 180)	43 (23.9)	51 (28.3)	86 (47.8)	*p* = 0.2740

## Perceptions on security – Further analysis

### Perceptions on efficiency of security personnel

The analysis was expanded to investigate possible differences in the main HCPs categories (doctors and nurses) and gender differences.

Table 3 has shown that the perceptions of all HCPs on the efficiency of hospital security personnel yielded a statistically significant difference between those who agreed and those who disagreed (*p* < 0.0001). However, when the perceptions of the main categories of HCPs (doctor and nurse) on the efficiency of hospital security personnel were compared, there was no significant difference among those who affirmed and those who did not, between the two groups (*p* > 0.05). However, more doctors (6; 28.6%) than nurses (14; 10.1%) indicated that they did not know about the efficiency of the hospital security system (*p* = 0.0174). Comparison of the perceptions of HCPs on the efficiency of the hospital security personnel by gender did not yield a statistically significant difference (*p* > 0.1000) in all the options (Box 1).

BOX 1 Comparison of perceptions on hospital security efficiency by health care professionals category (doctors and nurses) and gender.

**Table:** 

The hospital security system is efficient, *n* (%)			
	Doctor	Nurse	*P*
Agree	11 (52.4)	78 (56.5)	0.7252
Disagree	4 (19.0)	46 (33.3)	0.1898
Do not know	6 (28.6)	14 (10.1)	0.0174
Total	21 (100.0)	138 (100.0)	
	**Male**	**Female**	***P***
Agree	21 (46.7)	76 (56.7)	0.2454
Disagree	15 (33.3)	43 (32.1)	0.8820
Do not know	9 (20.0)	15 (11.2)	0.1350
**Total**	**45 (100)**	**134 (100)**	
**Reported security incidents are dealt with efficiently,** ***n* (%)**			
	**Doctor**	**Nurse**	***P***
Agree	3 (14.3)	39 (28.1)	0.1819
Disagree	9 (42.9)	33 (23.7)	0.0631
Do not know	9 (42.9)	67 (48.2)	0.6513
Total	21 (100)	139 (100)	
**The incident reporting system to the relevant authorities is efficient,** ***n* (%)**			
	**Male**	**Female**	***P***
Agree	21 (46)	46 (33.8)	0.1425
Disagree	16 (35.6)	41 (30.1)	0.4922
Do not know	8 (17.8)	49 (36)	0.0231
Total	45 (100)	136 (100)	

There was no significant difference between the perceptions of doctors and nurses regarding the efficiency in which reported security incidents were attended to (*p* > 0.05; Box 1). Although there was also no significant difference in the perceptions of male and female HCPs on the efficiency of the incident reporting system to the relevant authorities, there were significantly more female HCPs who indicated that they did not know about this efficiency (*p* = 0.0231; Box 1).

### Perceptions on safety

Table 4 shows that the hospital infrastructure, surroundings, safety from fire hazards, emergency evacuation plan and confidence to follow the latter were perceived as safe (*p* < 0.0001). The hospital lighting system was perceived as inadequate (*p* = 0.0041). Almost an equal proportion of HCPs had affirmative and dissenting perceptions on their safety from possible harm from patients (81; 45.5% vs. 89; 50.0%; *p* = 0.4614) and their visitors (78; 43.8% vs. 85; 47.8%; *p* = 0.4708). It is noteworthy that 59.0% of HCPs were not aware of the protocol on violence prevention in the hospital and that among those who were aware of it there was a significant difference between those with a negative perception (49; 28.3%) versus those with a positive perception (22; 12.7%), *p* = 0.0008. Only 36 (20.2%) HCPs had the perception that the hospital authorities cared about their safety, which was significantly different from those who had the opposite perception (*p* < 0.0001).

**TABLE 4:** Perceptions of health care professionals on safety.

Perception	Agree *n* (%)	Disagree *n* (%)	Do not know *n* (%)	Agree versus disagree *P*-value
Hospital infrastructure and surroundings are safe (*n* = 178)	112 (63.0)	46 (25.8)	20 (11.2)	< 0.0001
The hospital environment is safe from fire (*n* = 177)	98 (55.4)	36 (20.3)	43 (24.3)	< 0.000
The hospital lighting is adequate to ensure safety (*n* = 180)	68 (37.8)	93 (51.7)	19 (10.6)	0.0041
The emergency evacuation plan is clear (*n* = 179)	102 (57.0)	33 (18.4)	44 (25.0)	< 0.0001
Confidence to follow the emergency evacuation plan in emergencies (*n* = 179)	100 (55.9)	43 (24.0)	36 (20.1)	< 0.0001
Safety from possible harm from patients in the hospital (*n* = 178)	81 (45.5)	89 (50.0)	8 (04.5)	0.4614
Safety from possible harm from patients’ visitors (*n* = 178)	78 (43.8)	85 (47.8)	15 (08.4)	0.4708
The protocol on violence prevention in the hospital (*n* = 173)	22 (12.7)	49 (28.3)	102 (59.0)	0.0008
The hospital authorities’ concern for employees’ safety (*n* = 178)	36 (20.2)	91 (51.1)	51 (28.7)	< 0.0001

### Perceptions on safety – Further analysis

As was done with respect to security matters, further analysis was also conducted on safety matters to investigate possible differences in the main HCPs categories (doctors and nurses) as well as gender differences.

There was a statistically significant difference on perceptions between the proportions of male and female HCPs who disagreed that they were safe from patients as well as the patients’ visitors, with proportionately more males disagreeing in each case (Box 2).

BOX 2 Perceptions of health care professionals on their safety from patients and visitors and hospital emergency plan.

**Table:** 

HCPs are safe from patients, *n* (%)			
	Male	Female	*P*
Agree	15 (33.3)	66 (49.6)	0.0584
Disagree	29 (64.4)	60 (45.1)	0.0256
Do not know	1 (02.2)	7 (05.3)	0.3880
Total	45 (100)	133 (100)	
**HCPs are safe from patients’ visitors,** ***n* (%)**			
	**Male**	**Female**	***P***
Agree	15 (33.3)	63 (47.4)	0.1004
Disagree	28 (62.2)	57 (42.9)	0.0255
Do not know	2 (04.4)	13 (09.8)	0.2612
Total	45 (100)	133 (100)	
**There is an effective emergency evacuation plan in the hospital,** ***n* (%)**			
	**Doctor**	**Nurse**	***P***
Agree	9 (42.9)	85 (61.6)	0.1055
Disagree	2 (09.5)	27 (19.6)	0.2659
Do not know	10 (47.6)	26 (18.8)	0.0034
Total	21 (100)	138 (100)	
**I am confident to follow the emergency plan in case of an emergency,** ***n* (%)**			
	**Doctor**	**Nurse**	***P***
Agree	11 (52.4)	84 (60.9)	0.4607
Disagree	4 (19.0)	28 (20.3)	0.8903
Do not know	6 (28.6)	26 (18.8)	0.2980
Total	21 (100)	138 (100)	

Regarding the perception on procedures to be followed in an emergency situation, there were significantly more doctors than nurses who did not know about the emergency evacuation plan (*p* = 0.0034; Box 2). Evaluation of confidence to follow the emergency plan in case of an emergency yielded no difference between doctors and nurses (*p* > 0.05; Box 2).

## Discussion

This study describes the perceptions of HCPs on security in the district hospital, focussing on security personnel and their efficiency. It also assessed the HCPs’ perceptions on their safety with respect to the hospital infrastructure and surroundings, including available measures in case of emergencies, as well as perceptions on the hospital authorities’ care about the safety of the hospital staff in general. The majority of respondents were nurses (77.9%), a figure consistent with most articles related to perceptions of HCPs and HCWs.^3,12,18^ This is as a result of the dominant number of the nursing fraternity personnel in health care institutions – globally.^19^

## Perceptions on security

On the statement on whether the presence of security personnel made HCPs feel secure, this study showed that more than half of the respondents (101; 58%) affirmed that they felt secure, which is consistent with the study by Shaw in Cincinnati Children’s Hospital Medical Centre in the United States, where over half (101, 55.5%) of the respondents indicated that they experienced a sense of security when there were more hospital security personnel on duty.^17^ Slightly more than half of the respondents (54.2%) had a positive perception on the efficiency of the security personnel at the hospital. They indicated that the efficiency of the security personnel made them feel secure.^17^ This response could translate into the HCPs themselves rendering an efficient service in an environment where they feel secure.^20^ The hospital management should therefore maintain the security personnel presence and their operations in the hospital premises as these seem to engender the necessary perception of security among the HCPs.

There was no significant difference in the perceptions of doctors (52.4%) and nurses (56.5%) on the efficiency of the security system. Regarding the nurse proportion, our findings were similar to those by Rodriguez et al.,^12^ where about 54% of the nurses had trust in the efficacy of the security systems in comparison with other professionals in a Level III Hospital in Bogota, Colombia. The sex of the respondents did not influence their perceptions on the efficiency of the security personnel as the males (46.7%) and females (56.7%) affirmed this efficiency. These findings could not be compared with other studies because of paucity of research work on this topic. This means that nurses and doctors regardless of their sex had similar perceptions on the efficiency of the security personnel.

Almost one-third (31.5%) of the respondents indicated that they did not know about the security reporting system of the hospital. According to the study by Abdullah et al.,^10^ safety reporting was perceived as the most important element in employees’ occupational health safety practices. In combating workplace violence, Gillespie et al.,^21^ also emphasised the importance of a universal violence incident reporting system in a given institution. Therefore, this could be an indication of neglect by the hospital authorities to raise awareness among the staff on the reporting system for safety and security in the hospital.

The finding that close to about one-in-two (47.8%) HCPs indicated that they did not know whether security incidents in the hospital were dealt with efficiently, and that only about one-quarter (23.9%) held the perception that reported incidents were dealt with efficiently, that is, to their logical conclusion raises a red flag for the hospital management. Literature has shown that poor perception on how reported incidents of unsafety are dealt with leads to reluctance from staff to even initiate the process of incident reporting.^14^ The hospital authorities need to improve on this aspect by involving the HCPs’ leaders to ensure that proper feedback is given to the HCPs.

## Perceptions on safety

The majority of the respondents (63.0%) agreed with the statement that the hospital infrastructure and surroundings were a safe place to work in and that the environment was safe from fire (55.4%). None of these items could be compared with other studies because of scarcity of research in this field. One study conducted by Rodriguez et al.^12^ showed different results in that, among the nursing and administrative personnel, only 2.71% and 2.77%, respectively, trusted the safety of the hospital surroundings. Just above the average number of respondents (51.4%) indicated that the hospital lighting system was inadequate to ensure safety. Safety conferred by the lighting system in an establishment has been demonstrated by the study by Steinman which showed over 50% of respondents affirming that providing adequate lighting of a hospital had increased the feeling of safety among the staff members.^9^ The authorities should attend to the lighting system of the hospital to enhance infrastructural feelings of safety and security.

Less than half of the respondents indicated some concern about their safety from the patients utilising the hospital (45.5%) or from the hospital visitors, including the relatives (43.8%). However, neither result showed a statistically significant difference between those affirming and negating the statement. The study by Shaw demonstrated fewer respondents (26.8%) who were concerned or fearful of patients that could turn violent, with about the same number (26.8%) concerned about visitors who could turn violent.^17^ This implies that in the majority of cases, patients and their visitor were not perceived as posing a safety threat to the HCPs.

More than half of the HCPs did not know that there was a written workplace violence prevention protocol in the hospital (59.0%). This might indicate the need to raise awareness among the HPCs about the existence of a written workplace violence prevention protocol in the hospital. The Centres for Diseases Control and Prevention (CDC) advocated for the availability of administrative responsibility on occupational safety in institutions, with a safety committee and education programmes, protocols, training, immunisation and prevention of health-related hazards.^22^ Most of the respondents (55.9%) indicated that they knew about the procedure to be followed in the case of emergency evacuation in the hospital, and almost the same percentage of respondents (57.0%) affirmed the existence of the emergency evacuation plan. Gillespie et al.^21^ have shown that individual knowledge and skills in universal precautions is important in the prevention and reduction of health care workplace violence. The hospital authorities should take note and follow up the finding that significantly more nurses than doctors had a negative perception about the effectiveness of the emergency evacuation plan in the hospital.

More than half of the HCPs (51.1%) were of the perception that the hospital authorities were not concerned about their emotional health and physical well-being. According to Erickson, when management blames the employee for injuries and accidents, occupational health safety performance decreases.^23^ For that reason, organisational culture on safety and security is vital in determining the level at which employers and employees pitch the implementation of the best practice on health safety. One in two of the HCPs had the perception that the hospital authorities cared about their safety (51.1%). This could be an indictment which needs to be corrected by the hospital management because it has been shown that in institutions with a strong safety climate, workers suffer fewer accidents – not only because of the implementation of the safety programmes, but also because the very existence of these programmes indicates to employees the commitment of the authorities about the former’s safety.^24,25^ Efforts to curb workplace violence include policy, process and environmental changes and these should be routinely communicated to staff so that workers are always kept informed and develop the sense that they are valued by the employer.^17^

## Strengths and limitations

To our knowledge, this study is the first to investigate the perceptions of HCPs on their safety and security according to the National Core Standards for Health Establishments in South Africa. Although the study was a cross-sectional study in design which could not establish causal relationships, its findings could be used to generate hypotheses which, in turn could be tested through larger sample studies involving a number of settings. The measuring tool used in this study was created de novo by the research team with the assistance of a statistician. The pilot study that was conducted helped to reduce possible ambiguities of the questions. Self-reports and perceptions do not necessarily reflect the true state of affairs in an institution and may be prone to information bias. However, this assessment offers a premise upon which implementation of policies can be based when working towards the realisation of the national core standards in a health care establishment. This study employed a quantitative method and could not provide in-depth understanding of the HCPs’ views. In addition, the study was conducted in only one of the district hospitals in Gauteng and may not be a true representation of the perceptions of the entire health care workforce in all the district hospitals in the Gauteng province of South Africa. Future studies from more representative sites and employing qualitative methods are needed to fully investigate this topic.

## Conclusion

This study shows that perceptions of HCPs on security conferred by security personnel presence, efficiency of security personnel and efficiency of the security system were significantly affirmed. HCPs perceptions were positive but varied on issues of security personnel, security infrastructure, safety around patients, reporting system, emergency and evacuation plans. Negative perceptions regarding lighting in the hospital premises and management’s lack of concern for HCPs safety underscore the need for interventions to address these negative perceptions.
